# Microbial Invasion vs. Tick Immune Regulation

**DOI:** 10.3389/fcimb.2017.00390

**Published:** 2017-09-05

**Authors:** Daniel E. Sonenshine, Kevin R. Macaluso

**Affiliations:** ^1^Department of Biological Sciences, Old Dominion University Norfolk, VA, United States; ^2^Department of Pathobiological Sciences, Louisiana State University Baton Rouge, LA, United States

**Keywords:** pathobiology, Arp 23, caveolae, clathrin, salp 16, P11, JAKSTAT

## Abstract

Ticks transmit a greater variety of pathogenic agents that cause disease in humans and animals than any other haematophagous arthropod, including Lyme disease, Rocky Mountain spotted fever, human granulocytic anaplasmosis, babesiosis, tick-borne encephalitis, Crimean Congo haemorhagic fever, and many others (Gulia-Nuss et al., 2016). Although diverse explanations have been proposed to explain their remarkable vectorial capacity, among the most important are their blood feeding habit, their long term off-host survival, the diverse array of bioactive molecules that disrupt the host's natural hemostatic mechanisms, facilitate blood flow, pain inhibitors, and minimize inflammation to prevent immune rejection (Hajdušek et al., 2013). Moreover, the tick's unique intracellular digestive processes allow the midgut to provide a relatively permissive microenvironment for survival of invading microbes. Although tick-host-pathogen interactions have evolved over more than 300 million years (Barker and Murrell, 2008), few microbes have been able to overcome the tick's innate immune system, comprising both humoral and cellular processes that reject them. Similar to most eukaryotes, the signaling pathways that regulate the innate immune response, i.e., the Toll, IMD (Immunodeficiency) and JAK-STAT (Janus Kinase/ Signal Transducers and Activators of Transcription) also occur in ticks (Gulia-Nuss et al., 2016). Recognition of pathogen-associated molecular patterns (PAMPs) on the microbial surface triggers one or the other of these pathways. Consequently, ticks are able to mount an impressive array of humoral and cellular responses to microbial challenge, including anti-microbial peptides (AMPs), e.g., defensins, lysozymes, microplusins, etc., that directly kill, entrap or inhibit the invaders. Equally important are cellular processes, primarily phagocytosis, that capture, ingest, or encapsulate invading microbes, regulated by a primordial system of thioester-containing proteins, fibrinogen-related lectins and convertase factors (Hajdušek et al., 2013). Ticks also express reactive oxygen species (ROS) as well as glutathione-S-transferase, superoxide dismutase, heat shock proteins and even protease inhibitors that kill or inhibit microbes. Nevertheless, many tick-borne microorganisms are able to evade the tick's innate immune system and survive within the tick's body. The examples that follow describe some of the many different strategies that have evolved to enable ticks to transmit the agents of human and/or animal disease.

## Borrelia burgdorferi

*Borrelia burgdorferi* (sensu latu), the causative agent of Lyme disease, is a spirochete, a Gram-negative helically coiled bacterium approximately 0.5 μm wide by 20 μm long, with a flagellum below the outer membrane that controls its whiplash-like movements.

These bacteria are transmitted by ticks of the genus Ixodes, especially *I. scapularis* in North America and *I. ricinus* and *I. persulcatus* in Europe and Asia. In its tick host, *B. burgdorferi* is an intercellular pathogen, i.e., it survives in the midgut lumen, then migrates between the midgut's epithelial cells into the hemolymph and then into the salivary gland ducts. Bacteria are acquired by the tick host during blood feeding. Larval ticks ingest spirochetes while feeding on small mammals, especially white-footed mice. During and after feeding, the spirochetes remain within the midgut lumen. Those spirochetes trapped outside of the rapidly developing peritrophic membrane are unlikely to survive. Others between the membrane and midgut epithelial cells use an external surface lipoprotein, OspA, to bind to a species-specific receptor, TROSPA, located on the luminal surfaces of the midgut epithelial cells (Pal et al., 2004). Although surviving spirochetes may undergo an initial phase of multiplication, these populations decline during the tick's post-feeding molt cycle, perhaps due to antimicrobial effects of by-products of hemoglobin digestion, competition for nutrients and other unknown factors. Following molting to the nymphal stage, feeding by the recently molted nymph stimulates surviving spirochetes to begin prolific multiplication and attempt migration out of the midgut lumen (reviewed by Ogden et al., 2014) During this initial phase, the spirochetes form a complex network that moves between the epithelial cells toward their basolateral surfaces. OspA expression is reduced, allowing spirochetes to detach. Subsequently, they transition to the second phase of midgut migration, in which the spirochetes become motile, separate and escape through the basement membranes into the hemocoel (Dunham-Ems et al., 2009). Some host derived factors also play a role in the process, e.g., host-derived plasminogen, which protects the bacteria against phagocytosis and possibly even enhances their ability to penetrate the basement membrane (Coleman et al., 1997). Bacterial enolase was reported as the surface receptor that binds to the midgut receptor, Tre31, which facilitates migration out of the midgut (Zhang et al., 2011). It also binds host-derived plasminogen in the midgut and degrades it to plasmin (Noguiera et al., 2012). Borreliae upregulate OspC, which also binds and immobilizes plasmin, the enzymatically active form, which further enhances degrading intercellular matrices and other barriers, such as basement membranes (Önder et al., 2012). Nevertheless, the vast majority of spirochetes migrating into the hemolymph are destroyed, mostly by phagocytic hemocytes (Coleman et al., 1997). Upon contact with the salivary glands, borreliae bind to SALP15 (Ramamurthy et al., 2011), an immunosuppressive factor that protects these spirochetes from antibody-mediated killing, as well as other salivary gland proteins e.g., tick salivary lectin pathway (Schuijt et al., 2011) and tick histamine release factors (Dai et al., 2010) that also protect these spirochetes from host immune reactions (de Silva et al., 2009; Hajdušek et al., 2013). Exploitation of host-derived factors that enable *B. burgdorferi* to multiply and evade innate immune attack suggests that ticks tolerate these pathogens in the midgut but not in the hemolymph and other body tissues. Nevertheless, many questions remain, especially how the spirochetes are able to penetrate between the tightly bound midgut epithelial cells, avoid triggering expression or upregulation of antimicrobial peptides, and how they the penetrate salivary gland acini for dissemination into vertebrate hosts. Overarching factors directed by the tick microbiome (Narasimhan and Fikrig, 2015) will also be important when examining the infection of and transmission by ticks.

## Rickettsia rickettsii

Little is known about the specific infection mechanisms of spotted fever group (SFG) *Rickettsia* and their tick hosts (Munderloh and Kurtti, 1995, compared with the mammalian host cell. These bacteria, as well as other species of the Rickettsiales, invade host cells by binding to cellular receptors by means of their outer surface cell antigens (sca0 or rOmpAa and sca5 or rOmpB) and are internalized by receptor-mediated endocytosis via clathrin-coated vesicles, whereupon the microbes are incorporated into phagosomes (Chan et al., 2010). A similar sca5-mediated invasion mechanism used for vertebrate cells is employed by rickettsiae for invasion of tick cells (Thepparit et al., 2010). Upon invasion, rickettsiae quickly lyse these inclusions to escape into the cytosol. Once in the cytosol, rickettsiae replicate and then hijack the host cell's actin cytoskeleton and attach to it via actin tails (Gouin et al., 2005). The actin protein complex Arp2/3 is essential for the internalization of *R. rickettsii*, as well as other known SFG rickettsiae (Petchampai et al., 2014). In the vertebrate host cell, the bacteria express rickA, which promotes the activation of the host cell actin complex. This enables these bacteria to be propelled throughout the host cells as well as into protrusions that mediate cell to cell infection, thereby spreading the infection throughout the surrounding tissues (Gouin et al., 2004; Jeng et al., 2004). These actions are also effected by other cell proteins—profilin, fimbrin/T-plastin, capping protein, and cofilin, essential to actin assembly (Serio et al., 2010). In contrast to *R. rickettsii* which spread by means of actin bridges, *R. parkeri* and perhaps other rickettsial species, manipulate the intercellular tensions and mechano-transduction between host cells to facilitate their spread (Lampson et al., 2016). The roles of specific Sca molecules in facilitating rickettsial dissemination within the vector are under investigation. However, the host cell is not without a defense, as it is appreciated that ticks respond to rickettsiae (Macaluso et al., 2003; Mulenga et al., 2003). Using a tick cell culture (ISE6), investigators observed that pathogen infection led to decreased glucose metabolism but increased subolesin and heat shock protein expression, limiting rickettsial infection (Gillespie et al., 2012).

## Anaplasma phagocytophilum

**Figure 1:**
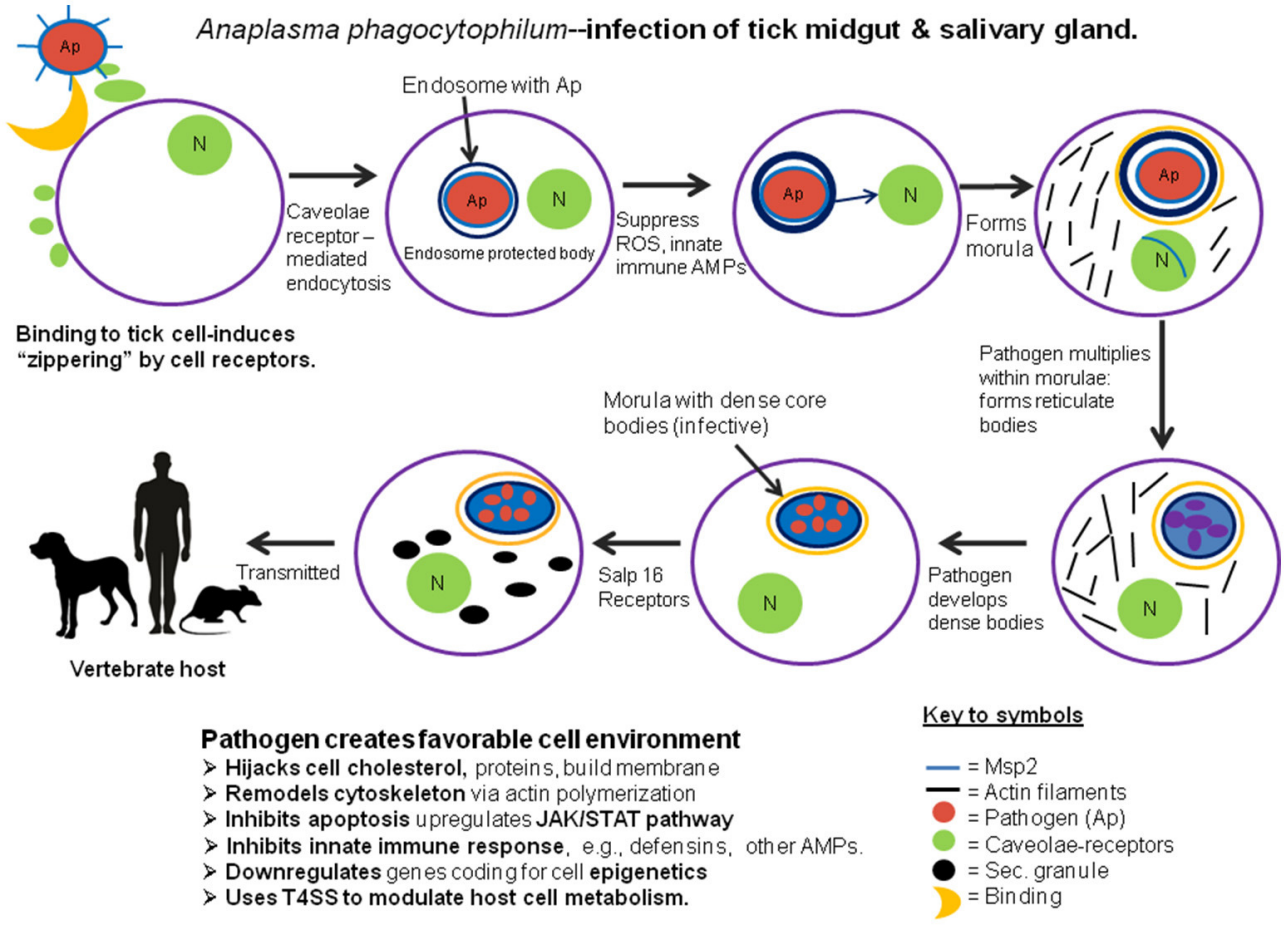
Diagrammatic representation of the process of invasion and development of the pathogenic microbe, *Anaplasma phagocytophilum* in the midgut and salivary glands of its tick host.

These bacteria employ a novel strategy for invading their host cells, evading cellular killing actions, manipulating the host cell's molecular machinery, and creating protected enclosures for their development and multiplication. In the tick, as well as in their vertebrate hosts, these bacteria avoid recognition by the host's innate immune system because they lack either peptidoglycans or lipopolysaccharides in their cell walls (Rikihisa, 2010). *A. phagocytophilum* bacteria also avoid the clathrin-receptor mediated endocytosis and phagolysosome typically used by host cells to capture and destroy invasive microbes. Instead, these bacteria are internalized via caveolae-mediated endocytosis: bacteria interact with caveolae and glycosylphosphatidylinositol-anchored proteins which enables them to bypass conventional phagolysosomes and form specialized endosomes. Bacterial outer surface protein MSP2 induces host intracellular signaling via an extracellular stimulation membrane receptor which induces recruitment of endocytic machinery at the binding site. This response leads to “zippering” around the pathogen and internalizing the microbe inside the host cell (Ireton, 2013). In tick cell cultures, *A. phagocytophilum* adhere to the cell membranes within 40 min post-infection. After binding, bacteria invade the host cells and form the specialized membrane-bound enclosures known as morulae. A mutant form of O-methyltransferase, identified as Msp4, also facilitates infection of the tick cells by *A. phagocytophilum* (Oliva Chávez et al., 2015). Within morulae, bacteria downregulate NADPH expression in enclosures, thereby minimizing reactive oxygen species (ROS) by suppressing expression of glutathione-s-transferase, superoxide dismutase and heat shock proteins (IJdo and Mueller, 2004). Like other rickettsiae, *A. phagocytophilum* alters cell gene expression (spectrin, fodrin) to control actin synthesis and remodel the host cell's cytoskeleton (de la Fuente et al., 2016). In mammalian cells, *A. phagocytophilum* hijacks host cholesterol to use in building the membrane surrounding the morulae (Xiong et al., 2009); however, whether this also occurs in tick cells is not known. In addition to these common strategies for infecting their hosts, *A. phagocytophilum* also exhibit more selective responses to different tissues. To infect the tick's midgut epithelial cells, these bacteria express genes that upregulate the JAK/STAT pathway, thereby inhibiting cell apoptosis (Ayllón et al., 2015). In addition, Cabezas-Cruz et al. (2016) suggest that *A. phagocytophilum* manipulates the host cell's epigenetics, increasing expression of histone deacetylase, Sirtuin and other molecules, which inhibits apoptosis and facilitates the microbe's multiplication. In addition, *A. phagocytophilum* infection can increase the levels of the tick's histone-modifying enzymes which makes it possible to regulate transcription and apoptosis selectively in different tissues, thereby not only facilitating both the pathogen's and the tick's survival. After escaping from the midgut, a salivary protein, P11, enables the microbes to infect circulating hemocytes, thereby enabling their migration to the salivary glands (Liu et al., 2011). Upon contact of infected hemocytes with the salivary gland cells, they induce expression of the salivary gland gene salp16 (Sukumaran et al., 2006), facilitating binding to the target cells. Following invasion of these host cells, the bacteria suppress the apoptotic mechanism by downregulating host cell porin expression, resulting in inhibition of cytochrome C release and thereby enabling their survival in the salivary glands. By upregulating this gene, the bacteria are able to selectively regulate transcription of this gene in association with RNAPII and the TATA-binding protein. However, the tick host is not without defenses. Recent work by Shaw et al. (2017), demonstrates a role for the tick IMD pathway in restricting *A. phagocytophilum* colonization of *I. scapularis*. Likewise, to control infection, tick salivary gland cells may limit *A. phagocytophilum* infection by inducing apoptosis via the extrinsic apoptosis pathway. Thus, in contrast to the midgut, these bacteria had considerably lower impact on salivary gland cells, presumably because they do not develop or multiply in that organ (Ayllón et al., 2015). Understanding the immune-related factors coordinating the balance between restriction and colonization of the vector is central to understanding vector competence. An illustration of how these pathogenic bacteria invade the tick host cells, multiply and prepare for transmission to their vertebrate hosts is shown in Figure 1.

## Babesia microti

This eukaryotic microbe is the causative agent of human babesiosis, and is closely related to similar protozoans that cause deadly febrile disease in cattle and other livestock throughout the world. *B. microti* parasites are transmitted to humans during blood feeding of its vector, *I. scapularis*. After inoculation into the human host, the parasites (in the form of sporozoites) invade and undergo their development in the erythrocytes. These apicomplexan parasites express a membrane protein, apical membrane antigen 1 (AMA1), located near its apical end of its cell body that binds to the surfaces of the red cells, facilitating invasion (Moitra et al., 2015). Within the erythrocytes, the parasites transform into trophozoites (vegetative stage), divide into 2–4 merozoites. The merozoites lyse the host cells, escape into the blood plasma and invade other red blood cells. In their new host red blood cells, some mature into gametocytes. During tick blood feeding, the ingested erythrocytes are lysed in the tick's midgut lumen, liberating the *B. microti* gametocytes. The latter give rise to gametes, some of which develop a spike-like arrowhead organelle. These arrowhead-bearing gametes are known as strahlenkorpers. These gametes fuse to become zygotes. Zygotes metamorphose into elongated, motile 8–10 μm parasites, which proceed to invade the tick's midgut epithelial cells. To penetrate the peritrophic membrane, *B. microti* use their spike-like arrowhead organelles to rupture the membrane and allow these microbes to cross the membrane and access the lining epithelial cells. Upon contact with the midgut cells, contact by the arrowheads induces the membranes to invaginate around the babesias and allows them to invade the host cells (Rudzinska et al., 1979). Once inside the midgut cells, the arrowhead organelle is lysed and disappears (Rudzinska et al., 1983). Little is known about the life cycle of the parasite within the tick tissues, especially how they suppress or evade recognition by the tick's immune system in order to develop within the midgut cells. Ultimately, the parasites emerge from the midgut epithelium, transform into motile kinetes that escape into the hemolymph and invade the tick's salivary glands. Following invasion of the salivary glands, the microbes transform into sporoblasts. Development is arrested until the tick, usually a nymph, feeds again, whereupon thousands of sporozoites are produced from each sporoblast. The sporozoites are the infectious stage for the vertebrate host. Sporozoites are transmitted to the new host during tick feeding, whereupon they invade and develop within the host's erythrocytes.

## Author contributions

DS wrote the preliminary draft. KM edited the draft and contributed additional information and related references. DS designed the original figure. KM revised the figure to emphasize clarity of the infection process and progress toward transmission, as well as improving the image quality. Both authors edited and accepted the final draft.

### Conflict of interest statement

The authors declare that the research was conducted in the absence of any commercial or financial relationships that could be construed as a potential conflict of interest.
